# Iron Deficiency Anemia as a Factor in Male Infertility: Awareness in Health College Students in the Jazan Region of Saudi Arabia

**DOI:** 10.3390/ijerph182412866

**Published:** 2021-12-07

**Authors:** Mohammad S. Akhter, Hassan A. Hamali, Johar Iqbal, Abdullah A. Mobarki, Hina Rashid, Gasim Dobie, Aymen M. Madkhali, Bader Y. H Arishi, Emad O. O. Ageeli, Osama S. H. Laghbi

**Affiliations:** 1 Department of Medical Laboratory Technology, Faculty of Applied Medical Sciences, Jazan University, Jizan 45142, Saudi Arabia; hhamali@jazanu.edu.sa (H.A.H.); abMobarki@jazanu.edu.sa (A.A.M.); gdobie@jazanu.edu.sa (G.D.); ammadkhali@jazanu.edu.sa (A.M.M.); B3rishi@gmail.com (B.Y.H.A.); emad.ageely@gmail.com (E.O.O.A.); posama1008@gmail.com (O.S.H.L.); 2 Department of Biochemistry, College of Medicine, Jazan University, Jizan 45142, Saudi Arabia; jauhariqbal@gmail.com; 3 Department of Pharmacology and Toxicology, College of Pharmacy, Jazan University, Jizan 45142, Saudi Arabia; hzehqeer@jazanu.edu.sa

**Keywords:** male infertility, iron, micronutrient, iron deficiency anemia

## Abstract

Male contribution towards couple infertility is increasing but is less discussed. We aimed to assess the knowledge about iron deficiency anemia (IDA) as a contributor to male infertility in students at health colleges of Jazan University. A multicentric, cross-sectional survey included 910 participants and 768 participants qualified as per our inclusion criteria. The questions were categorized as: Model 1—knowledge about IDA-induced male infertility; Model 2—knowledge about IDA. The average knowledge of IDA causing male infertility is very low among students. The 18–20 years age group had a lesser score for either knowledge of IDA (M2; *p*-value = 0.047) or total (*p*-value < 0.0001) compared to the older group. In addition, female students were significantly more likely to be better in achieving higher total scores (*p*-value = 0.023) as well as M2 scores (*p*-value < 0.0001) when compared to the respective male category. On the other hand, males were significantly better in scoring for M1 (*p*-value = 0.004) compared to females. Awareness about iron deficiency anemia as a factor in male infertility may reduce the infertility burden, arising from a preventable factor, in the Jazan region.

## 1. Introduction

Infertility is an unceasing reproductive condition that is well-recognized by the World Health Organization and its prevalence has increased over the last few decades. The condition affects both men and women and is most commonly self-diagnosed by an inability to conceive [1]. Infertility is associated with increased adverse mental and social consequences, resulting in anxiety, depression, and even divorce [2,3]. While infertility has often been considered a female condition with male contribution overlooked, both males and females can contribute towards it. It is now well known that 30% of infertility cases can be ascribed solely to females, whilst 30% of cases can be ascribed entirely to males, 30% of cases can result as a contribution of both partners, and 10% of cases have an unknown cause [4]. Male infertility has gained considerable recognition since the 1970s as one of the strong contributing factors to couple infertility and as a predictor of mortality and the general health status of males [5]. However, there is still no accurate data available on the global incidence of male infertility because of cultural considerations and patriarchal societies around the globe [6]. In 70% of cases of male infertility the underlying etiology remains unclear, while the recognizable underlying causes can be genetic, epigenetic modifications caused by environmental toxins or drugs, lifestyle choices, medical illnesses or medications, and nutritional state [7,8,9]. Many of these causes of male infertility may be treatable or preventable and require an accurate assessment of the condition and identification of the cause to prevent further impairment in fertility [10]. Numerous testicular factors, gonadotoxins, and lifestyle factors have been studied as preventable factors of male infertility and the health care providers and educators tend to inform the general public and the vulnerable groups about the reproductive risks associated with such factors, e.g., smoking, alcohol, etc. However, less attention has been given to the nutritional state as a preventable contributing factor to male infertility. Vitamins, minerals, and micronutrients are necessary for good reproductive health in males and their deficiencies can lead to impaired male fertility, but this aspect seems to be emphasized less by the health care system. It is reported that testicular physiology is sensitive to alterations in whole-body metabolism and micronutrients have a crucial role in maintaining a healthy metabolism [8,11]. Iron is one such nutrient that has a vital role in maintaining normal spermatogenesis and healthy male reproduction. A deficiency of iron may manifest as IDA and is an urgent public health problem [12]. IDA can be a result of multiple factors, most notably, iron and other nutrient deficiencies, malaria, non-specific inflammation, and genetic blood disorders. The global burden of IDA is mostly driven by females and children, but an increasing trend in males has been reported in numerous studies, most commonly resulting from chronic gastrointestinal blood loss and poor dietary choices [13,14,15]. IDA is known to occur in 2% to 5% of adult men and may have deleterious reproductive outcomes in them [16]. With dietary patterns quickly changing all over the world, from healthy to nutrition-deprived food, and a concurrent increase in stressful lifestyles, it becomes imperative to increase the awareness of males in general and vulnerable groups in particular of IDA to prevent impaired fertility arising from it. Since limited data is available for the effects of IDA on male fertility, the condition needs to be studied thoroughly and the general public should be made aware of its adverse reproductive consequences to prevent it from becoming a sizeable health condition. A continuous educational activity that places emphasis on the significance of various nutrients for healthy development and growth can have a positive effect on the general public as well as on those of reproductive ages and could decrease the burden of disease and dysfunction associated with nutritional deficiencies. Awareness programs and audience-specific educational programs can be of primary importance in making people aware of the lesser-known but precarious health outcomes. In this study, we aimed to assess the attitude and awareness of the students at health colleges in the Jazan region about IDA and its associated effects on male fertility. We expected the specific group to be more aware and informed about the influence of nutritional status on overall health; however, their knowledge about the association of IDA and male fertility was dubitable and required to should be assessed to help frame policies to increase awareness about nutrition and reproduction in males.

## 2. Materials and Methods

### 2.1. Study Design and Population

We designed a multicentric, cross-sectional survey to investigate the knowledge and awareness of IDA as a contributing factor to male infertility in health college students of Jazan University. The inclusion criterion was to assess only the undergraduate students at the health colleges (College of Applied Medical College (CAMS), College of Pharmacy (CP), College of Nursing (CN), College of Dentistry (CD)) of the Jazan University belonging to Jazan province, Saudi Arabia. The study was conducted from January 2021 to April 2021.

### 2.2. Data Collection

**Figure 1:**
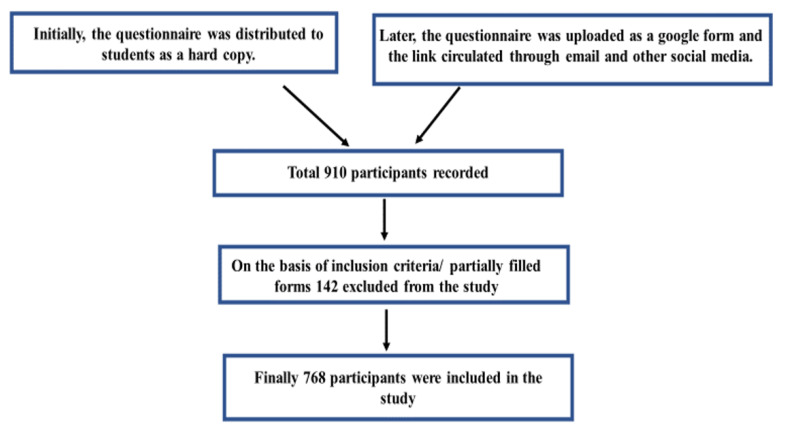
Flow chart of participant’s recruitment.

A self-designed, structured questionnaire was developed to assess the knowledge and attitude of the students about IDA and IDA as a contributing factor to male infertility. Initially, we distributed the questionnaire as hard copy among students and collected their responses, but later we made the questionnaire into a google form, for an easy approach and to increase the number of participants. The questionnaire was shared between students through email and through WhatsApp by the research team (Figure 1).

### 2.3. Scoring

Awareness and knowledge about IDA as a factor in male infertility was assessed through the questionnaire. The questionnaire comprised 20 closed-ended (Supplementary File S1) questions, which were divided into three sections. The first section comprised of four questions that focused on the demographic distribution and professional characteristics of the participants. This section collected information about: age, gender, dwelling area, and the educational discipline of the participants. The second and third sections included 16 questions that were designed to gather the participants’ knowledge about iron, IDA, and IDA as a factor in male infertility. For each question, if the respondents gave a ‘yes’ response, they scored 1 point; if they responded ‘no’ then the score was 0. Total scores thus ranged from 0 to 16. Then, we set a score to identify the status of respondents’ awareness and knowledge of IDA as a factor in male infertility. Respondents with scores ranging from 0 to 8 were considered to have poor awareness and knowledge, whereas those with scores ranging from 9 to 16 points were considered to have high awareness and knowledge.

### 2.4. Ethical Consideration

The study was approved by the Standing Committee for Scientific Research Ethics, Jazan University (HAPO-10-Z-001). The objective of the study was explained to all participants.

### 2.5. Statistical Analysis

Values are expressed as mean ± standard deviation (SD) and median, interquartile range (IQR). A non-parametric test, Mann–Whitney U, was used to determine the statistical difference between values. Binary logistic regression was performed for multivariable analysis of the association between students’ characteristics (age groups, gender, place of living, and college of registration) and survey question response. In addition, the association of students’ characteristics and knowledge scores of IDA (M2), IDA causing male infertility (M1), and total survey were analyzed using a generalized linear model. All models were a good fit and the chi-square test was used to quantify significance. All analysis was performed on SPSS statistical package version 21 for windows and Microsoft excel. Odds ratios (OR) and beta coefficients (B) were expressed with their 95% confidence intervals (CI), and *p*-values less than 0.05 were considered significant.

## 3. Results

As shown in Table 1, a total of 768 students responded to the survey. Most of them were males (nearly 70%) belonging to the 21 to 25 years age group (72%). Approximately two-thirds of the respondents were students from the College of Applied Medical Sciences (64%), followed by the College of Nursing (18%), and the College of Pharmacy (16%). Very few students, only 16 in number, were from the College of Dentistry. Interestingly, students residing in rural areas showed better participation in this survey when compared to students residing in urban areas.

**Figure 2:**
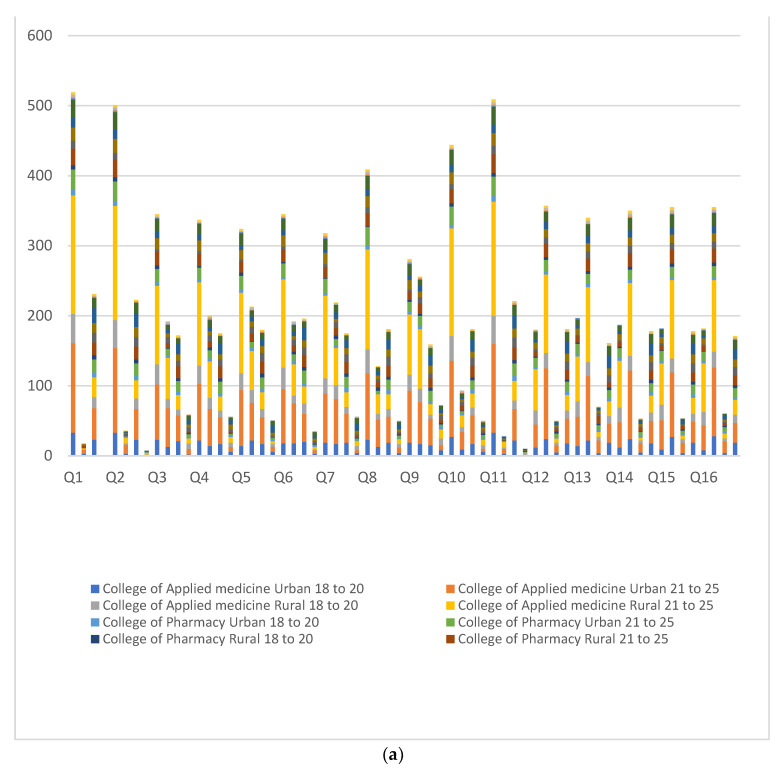

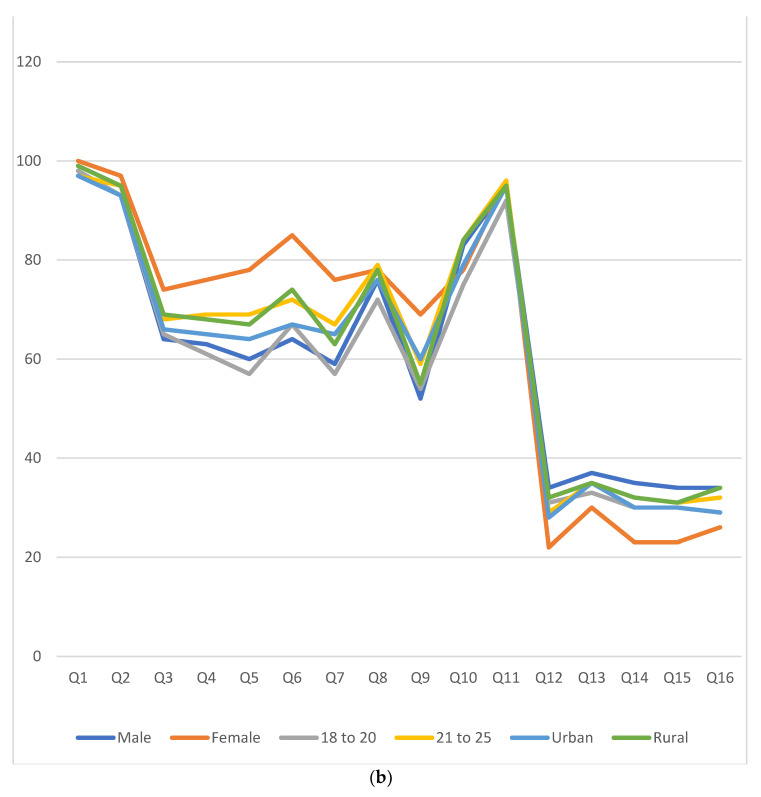
(**a**) Responses of the participants are depicted as stack bars in numbers. Four bars belong to each question. Responses as ‘yes’ are shown in the first and third bars from male and female participants, respectively. Similarly, the second and fourth bars represent a ‘no’ response. (**b**) Positive responses in percentage from various gender, age groups, and places of residence.

The survey response towards all 16 questions, concerning IDA and the effect of IDA on male infertility, is depicted in Figure 2a,b. As shown in these figures, females were better at answering ‘yes’ to question number (Q) 1 to Q11 (69 to 100%) in comparison to males in all categories, with a notable exception in Q10, where 83% of male students said ‘yes’ which is 5% higher than female respondents. On the other hand, most of the students (63 to 78%) had selected ‘no’ for Q12 to Q16 with a female preponderance in each question’s responses. In addition, the students living in the rural areas responded, ‘yes’ in greater strength (2 to 5%) to Q1 to Q6, Q8, Q10, Q12, and Q14 to Q16 as compared to respondents living in urban areas.

To determine the aggregate knowledge of students about IDA and its effect on male infertility, the questions were categorized into two groups, namely Model 1 (M1; knowledge regarding male infertility caused by IDA) and Model 2 (M2; knowledge concerning IDA). Table 2 shows that the average knowledge of IDA causing male infertility is very low among students (most of the students acquired a ‘0′ score) irrespective of the category. Additionally, the M1 and M2 scores were comparable in both the age groups in corresponding genders in the CAMS, the CP, and the CD, respectively. However, total and M2 scores were significantly lower (*p* = 0.014; 0.006) in the 18–20 years age group in comparison to older students.

To ascertain the predictive potential of students’ age, gender, college of education, and place of residence in answering ‘yes’ to survey questions, we performed multivariable binary logistic regression for each question. As shown in Table 3, the older age group was significantly more likely to answer ‘yes’ for Q4 to Q7 (OR/*p*-value; 0.68/0.027, 0.56/0.001, 0.68/0.039, 0.60/0.004) and Q9 to Q11 (OR/*p*-value; 0.69/0.032, 0.63/0.023, 0.43/0.015) when compared with younger age group students. Likewise, female students were significantly better in responding to Q3 to Q7 (OR/*p*-value; 1.71/0.003, 2.11/<0.0001, 2.76/<0.0001, 3.75/<0.0001, 2.52/<0.0001) and Q9 (OR/*p*-value; 2.09/<0.0001) in comparison to males. Conversely, in Q12, Q14, and Q15 (OR/*p*-value; 0.53/0.001, 0.56/0.002, 0.59/0.005), males were more likely to answer ‘yes’ in comparison to females. In addition, the analysis suggests that participants living in rural areas [Q6 (OR/*p*-value; 1.55/0.008)], studying in the CAMS [Q10 (OR/*p*-value; 3.07/0.036)] or the CP [Q15 (OR/*p*-value; 8.26/0.045)] were more likely to respond correctly to the respective questions when compared with their reference categories.

Further, to understand the association between students’ characteristics and their M1, M2, and total knowledge score, we performed multivariate regression analysis. As expected, the 18–20 years age group was significantly less likely to achieve a better score for either knowledge of IDA (M2; *p*-value = 0.047) or total (*p*-value < 0.0001) as compared to the older age group. In addition, female students were significantly more likely to be better in achieving higher total scores (*p*-value = 0.023) as well as M2 scores (*p*-value < 0.0001) when compared to the respective category. On the other hand, males were significantly better in scoring for M1 (*p*-value = 0.004).

## 4. Discussion

Anemia is a health burden in the Gulf countries, with the prevalence of IDA ranging from 25% to 35%, and 12.9% of males aged 11–19 years contributing to it [17,18]. In our previous study on anemia among Jazan university students, we found 4.7% of males had anemia, but the finding might be biased due to sampling in a specific population and low sample size [19]. IDA can lead to health complications, if left unattended, in both females and males. There are increasing studies that strongly indicate that poor reproductive outcomes are associated with IDA in males; however, the general public and the vulnerable groups are less aware of this fact [20,21].

To assess the awareness of IDA in various populations in Saudi Arabia, various surveys have been conducted that indicate a fair, moderate, or satisfactory level of awareness among various populations about IDA and associated risks especially in females, however, no study evaluates awareness about IDA and male infertility [22,23,24]. The current survey provides a perception about the cognizance and attitude of ancillary medical students in the Jazan province of Saudi Arabia about IDA as an important contributing factor to male infertility. We observed varied knowledge about various aspects of IDA, but a low cognition of IDA as a contributing factor to male infertility in such students (Table 3).

In our study, students from various colleges were assessed on their knowledge, awareness, and attitude about IDA as an important factor in male infertility. However, most of the respondents were males, and from the CAMS. This indicated a difference between the online proclivity among the two genders, that has been reported earlier by Smith et al. 2008 [25].

The greater participation by the CAMS may have resulted due to ease of outreach by the investigators and as a sense of belonging to the institution since affiliation is a well-recognized factor that affects the response to online surveys [26].

When nutritional aspects of iron were considered, females were more aware of iron as a micronutrient, the foods that are rich in iron, and they also recognized that IDA arises from iron deficiency (Table 3, Figure 2b). However, the cognition of association of male infertility with IDA was low for both genders, with females even less aware than males (Table 3). This may be a result of the lack of attention that the male contribution to couple infertility has received over the years. This indicates the need for more responsible behavior from the health care system to disseminate inconspicuous information about IDA and male fertility. IDA is one of the causes of male infertility that can be prevented or treated to improve reproductive outcomes and increased awareness about it may lead to more preventive measures by vulnerable groups. An adequate intake of iron by males is essential since iron is highly required to maintain ejaculate fluidity and sperm pH within a functional range [27,28]. In addition, Sertoli and Leydig cells are sources of ferritin for the developing sperm and protect testicular tissue [29,30]. IDA leads to reduced circulatory oxygen transport creating a hypoxic environment to the testes, which impairs spermatogenesis [31]. In a pilot study by Soliman et al. 2014, they reported that correction of IDA was associated with significant enhancement of sperm parameters and increased concentrations of serum LH, FSH, and testosterone in eugonadal males with iron-deficiency IDA [32]. Correction of IDA improves hypoxia and subsequently elevates gonadotropin hormones [33]. These studies indicate the importance of iron for male reproduction; however, less awareness about these aspects in our study population is a concern since the heath college students should have a higher level of awareness about nutrition and various health aspects and they represent young adults entering their reproductive years.

The level of education seemed to affect the knowledge since older students in higher-level semesters were more aware of IDA when compared to younger students (Table 4). It reflects that the knowledge and awareness levels of higher semester students are better than low semester students for IDA, possibly arising out of the advanced curriculum and knowledge gained from peers and family.

Students from rural areas had increased participation and knowledge compared to those from urban areas; this was a noteworthy point, possibly indicating a higher level of inquisitiveness. This is also in agreement with the observations of Rayfield et al. (2008) and Croft and Moore (2019), who observed that rural students participate more in extracurricular activities than their urban counterparts [34,35].

The most significant finding from the survey was that the students had a very low level of knowledge and awareness about IDA as a contributing factor to male infertility and the associated biochemical mechanisms underlying this effect, irrespective of any demographic or gender factor. This is an important aspect that requires attention from health educators both in the educational sector as well as in the public domain to increase awareness about adverse reproductive effects in males.

Overall, it was evident from the results that most of the students were fairly aware of most of the aspects of IDA, but the majority of the students were not aware of the effects of IDA on male reproduction. This is mainly because there is limited data and discourse on IDA-induced male infertility which may lead to low awareness among communities.

These findings are significant for the general public all over the world and specifically for the people of Jazan since it is an area with one of the highest incidences of hemoglobinopathies, mainly sickle cell anemia, in the Kingdom of Saudi Arabia. In patients with sickle cell anemia, abnormalities of semen volume, sperm morphology, and sperm motility are often found, which can lead to poor reproductive outcomes [36]. Preventing IDA in such patients can be significant in reducing the disease burden and improving the reproductive indices. Correction of IDA is associated with gonadotropin hormones levels and sperm parameters in sickle cell disease [37]. Similarly, in thalassemia major patients, improvement in hormone levels (serum FSH, LH, testosterone) and sperm parameters (sperm count and sperm morphology) were significantly associated with an increase in hemoglobin after packed red cell transfusion [38].

The prevention of IDA must be addressed to reduce the disease burden associated with iron deficiency in both genders and especially in the reproductively vulnerable groups. Since male infertility is receiving considerable recognition lately, awareness about IDA and male infertility should be made common. Students from the health colleges can be an important interface between society and the health care sector and they can provide the requisite awareness at various levels. However, low awareness about IDA and male infertility amongst the students should be addressed and rectified to help them attain their societal goals. Our study will increase knowledge and awareness about male infertility caused by IDA which can lead to the prevention of impaired male fertility arising from IDA.

We affirm to the best of our knowledge that this is the first study of its type to assess the awareness of IDA and male infertility based on PubMed and Google Scholar database exploration.

## 5. Conclusions

IDA as a contributing factor to male infertility is poorly recognized by the students at health colleges in Jazan, which indicates the poor attention given to the contributing factors of male infertility in the educational and health sectors. These findings may also be important for other populations where IDA is a health issue and suggest that attention must be paid to improve the outlook towards IDA and male infertility.

## 6. Limitations

Although this is the first study of its type to explore awareness about IDA-induced male infertility, this study does have some limitations. First, it is a monocentric study that limits the generalization of our finding to other regions. Second, the study is conducted in health colleges only. Third, most of the respondents are from the CAMS. Fourth, the ratio of male and female respondents is not balanced.

## Figures and Tables

**Table 1:** Demographic characteristics of the respondents of the survey (N = 768).

Particulars	Characteristics	N (%)
**Gender**	Female	231 (30.1)
	Male	537 (69.9)
**Age (years)**	18 to 20	211 (27.5)
	21 to 25	557 (72.5)
**Place of Residence**	Urban	368 (47.9)
	Rural	400 (52.1)
**College of Registration**	College of Applied Medicine Science (CAMS)	495 (64.5)
	College of Nursing (CN)	137 (17.8)
	College of Pharmacy (CP)	120 (15.6)
	College of Dentistry (CD)	16 (2.1)

Values are presented as numbers and (percentage).

**Table 2:** Knowledge scores of various categories of students for IDA (M2), IDA causing male infertility (M1), and Total.

College	Gender	Age Group (N)	Age	Total	M1	M2
			Mean (SD)	Median (IQR)	Median (IQR)	Median (IQR)
**Applied Medical Science**	Male	18 to 20 (78)	19.2 (0.71)	10 (9.5)	1 (4.7)	8.5 (5)
		21 to 25 (305)	22.4 (1.21)	10 (6)	0 (4)	9 (4)
	Female	18 to 20 (39)	19.2 (0.68)	10 (3)	0 (1)	9 (2)
		21 to 25 (73)	22.6 (1.39)	11 (4)	0 (2)	10 (2)
**Nursing**	Male	18 to 20 (26)	19.2 (0.75)	6 (7) *	0 (1)	6 (4.7) **
		21 to 25 (47)	22.5 (1.28)	10 (7.5)	0 (4.5)	10 (5)
	Female	18 to 20 (35)	19.1 (0.80)	9 (3)	0 (1.5)	9 (2.5)
		21 to 25 (29)	22.4 (1.45)	9 (4)	0 (1)	8 (3)
**Pharmacy**	Male	18 to 20 (14)	19.1 (0.73)	8 (6.5)	0.5 (4.5)	7 (2.7)
		21 to 25 (56)	22.8 (1.22)	10 (7.2)	0 (5)	9 (3)
	Female	18 to 20 (13)	19.3 (0.75)	11 (5.5)	0 (4.2)	9 (3.2)
		21 to 25 (37)	22.4 (1.21)	11 (2)	1 (3)	11 (2)
**Dentistry**	Male	18 to 20 (5)	19 (0.70)	7 (3)	1 (2)	6 (3)
		21 to 25 (6)	22.7 (0.81)	10.5 (4)	0 (0)	10.5 (4)
	Female	18 to 20 (1)	20 (-)	5 (-)	0 (-)	5 (-)
		21 to 25 (4)	22.7 (2.06)	11 (0.75)	0 (0.7)	10 (2)

Values are presented as either mean (SD) or median (IQR). Values are significantly different from 21–25 years at * *p* < 0.05 and ** *p* < 0.01.

**Table 3:** Multivariable logistic regression for the association of students’ characteristics and respective survey questions.

Question	18–20 ^a^	Female ^b^	Rural ^c^	CAMS ^d^	CN ^d^	CP ^d^
1	1.16 (0.36–3.68)	6.24	2.50 (0.92–6.79)	3.84 (0.433–34.06)	2.60 (0.24–28.23)	2.72 (0.25–30.09)
2	0.80 (0.40–1.59)	2.05 (0.92–4.57)	1.40 (0.75–2.59)	2.35 (0.50–11.02)	2.96 (0.54–16.33)	2.61 (0.47–14.50)
3	0.85 (0.60–1.20)	1.71 ** (1.19–2.45)	1.18 (0.87–1.60)	1.26 (0.44–3.57)	0.99 (0.34–2.93)	1.73 (0.57–5.24)
4	0.68 * (0.48–0.96)	2.11 *** (1.46–3.04)	1.23 (0.91–1.68)	1.70 (0.60–4.77)	1.25 (0.43–3.67)	1.70 (0.57–5.07)
5	0.56 *** (0.39–0.79)	2.76 *** (1.89–4.03)	1.21 (0.89–1.64)	1.18 (0.40–3.47)	0.79 (0.26–2.40)	1.54 (0.49–4.80)
6	0.68 * (0.47–0.98)	3.75 *** (2.46–5.71)	1.55 ** (1.12–2.14)	1.65 (0.56–4.89)	1.09 (0.35–3.37)	1.54 (0.49–4.85)
7	0.60 ** (0.43–0.85)	2.52 *** (1.74–3.64)	0.98 (0.72–1.32)	0.83 (0.27–2.52)	0.53 (0.17–1.67)	0.90 (0.28–2.89)
8	0.71 (0.49–1.04)	1.24 (0.84–1.83)	1.13 (0.81–1.58)	0.79 (0.22–2.83)	0.65 (0.17–2.45)	0.76 (0.20–2.90)
9	0.69 * (0.50–0.97)	2.09 *** (1.49–2.95)	0.83 (0.62–1.11)	0.41 (0.13–1.31)	0.44 (0.13–1.48)	0.39 (0.12–1.32)
10	0.63 * (0.42–0.94)	0.88 (0.59–1.32)	1.35 (0.93–1.96)	3.07 * (1.08–8.78)	2.21 (0.74–6.63)	2.47 (0.81–7.57)
11	0.43 * (0.22–0.85)	1.38 (0.64–2.98)	1.00 (0.52–1.94)	1.16 (0.14–9.32)	1.11 (0.13–9.77)	1.68 (0.17–16.47)
12	1.24 (0.87–1.77)	0.53 *** (0.36–0.76)	1.17 (0.85–1.60)	1.28 (0.40–4.10)	1.21 (0.36–4.06)	1.70 (0.51–5.69)
13	1.03 (0.73–1.45)	0.76 (0.54–1.07)	1.01 (0.74–1.36)	2.35 (0.66–8.38)	1.61 (0.43–5.99)	3.44 (0.93–12.74)
14	1.10 (0.76–1.56)	0.56 ** (0.39–0.81)	1.08 (0.79–1.48)	3.35 (0.75–15.03)	2.64 (0.57–12.29)	4.00 (0.86–18.63)
15	1.15 (0.80–1.64)	0.59 ** (0.41–0.85)	1.05 (0.77–1.44)	7.18 (0.94–55.13)	5.17 (0.65–40.88)	8.26 * (1.05–65.19)
16	0.94 (0.66–1.34)	0.72 (0.51–1.03)	1.20 (0.88–1.64)	2.13 (0.59–7.60)	1.60 (0.43–5.96)	2.30 (0.62–8.58)

Values are presented as OR at 95% CI. Reference category ^a^ 21–25; ^b^ Male; ^c^ Urban; ^d^ CD. * *p* < 0.05; ** *p* < 0.01; *** *p* < 0.001.

**Table 4:** Generalized linear model for the association of students’ characteristics and their knowledge scores of various models.

Characteristics	Total				M1				M2			
	B	Upper	Lower	*p*-Value	B	Upper	Lower	*p*-Value	B	Upper	Lower	*p*-Value
**18–20 ^a^**	−0.629	−1.251	−0.008	0.047	0.082	−0.248	0.412	0.625	−0.711	−1.106	−0.317	<0.0001
**Female ^b^**	0.708	0.097	1.319	0.023	−0.476	−0.800	−0.151	0.004	1.184	0.795	1.572	<0.0001
**CAMS ^d^**	1.201	−0.702	3.105	0.216	0.804	−0.207	1.814	0.119	0.397	−0.813	1.607	0.520
**CN ^d^**	0.483	−1.499	2.464	0.633	0.542	−0.510	1.594	0.312	−0.059	−1.319	1.200	0.926
**CP ^d^**	1.501	−0.494	3.497	0.140	1.034	−0.025	2.094	0.056	0.467	−0.802	1.735	0.471
**Rural ^c^**	0.367	−0.176	0.910	0.185	0.101	−0.188	0.389	0.493	0.266	−0.079	0.612	0.130

Values are presented as unstandardized beta coefficient at 95% CI. Reference category ^a^ 21–25; ^b^ Male; ^c^ Urban; ^d^ CD.

## Data Availability

The data presented in this study are available on request from the corresponding author. The data are not publicly available due to ethical considerations.

## Supplementary Materials

The following are available online at https://www.mdpi.com/article/10.3390/ijerph182412866/s1, Questionnaire 1: Questionnaire to assess awareness about iron deficiency anemia as a factor in male infertility, in health college students in the Jazan region of Saudi Arabia.
