# Supplementary material for: Iron Deficiency Anemia as a Factor in Male Infertility: Awareness in Health College Students in the Jazan Region of Saudi Arabia
Source: Int J Environ Res Public Health. 2021 Dec 7;18(24):12866. doi: 10.3390/ijerph182412866 (PMC8701172; doi:10.3390/ijerph182412866)
Supplement: Supplementary file 1 [file ijerph-18-12866-s001.zip › File S1.pdf]

	Question	Response	
	Age		
	Gender		
	College		
	Where do you live, village, town, city		
		yes	No
	Do you know what is iron?		
	Do you know iron is a micro nutrient?		
	Do you know diet contains iron?		
	Do you know what foods contain iron?		
	Do you know what is iron deficiency anemia?		
	Do you know iron deficiency is one of the most common disorders affecting humans?		
	Do you know iron deficiency anemia can cause a lot of bad health effects?		
	Do you know iron deficiency anemia continues to be a major public health problem worldwide?		
	Do you know men can have iron deficiency anemia?		
	Do you know iron deficiency anemia can affect male fertility?		
	Do you know iron deficiency anemia imposes a hypoxic environment in different organs and tissues including the testes?		
	Do you know iron deficiency anemia can disrupt normal spermatozoa production?		
	Do you know iron deficiency anemia decreases sperm count and sperm motility in semen?		
	Do you know iron deficiency anemia makes germ cells more susceptible to damage?		
	Have you ever got checked for anemia?		
	Do you think importance of anemia in males needs to be addressed more?		
	Do you think awareness about male anemia should be increased in our community?		
